# Associations of Prevalence of E-cigarette Use With Quit Attempts, Quit Success, Use of Smoking Cessation Medication, and the Overall Quit Rate Among Smokers in England: A Time-Series Analysis of Population Trends 2007–2022

**DOI:** 10.1093/ntr/ntae007

**Published:** 2024-01-12

**Authors:** Sarah E Jackson, Jamie Brown, Emma Beard

**Affiliations:** Department of Behavioural Science and Health, University College London, London, UK; SPECTRUM Consortium, Edinburgh, UK; Department of Behavioural Science and Health, University College London, London, UK; SPECTRUM Consortium, Edinburgh, UK; Department of Behavioural Science and Health, University College London, London, UK; SPECTRUM Consortium, Edinburgh, UK

## Abstract

**Introduction:**

This study aimed to (1) provide up-to-date estimates of how changes in the prevalence of e-cigarette use have been associated with changes in smoking cessation activities and use of licensed treatments among smokers in England and (2) explore any changes in these associations over time.

**Methods:**

Data were aggregated quarterly on 67 548 past-year smokers between Q1-2007 and Q4-2022. Explanatory variables were the prevalence of (1) current e-cigarette use among smokers and (2) e-cigarette use during a quit attempt. Outcomes were rates of quit attempts and overall quits among past-year smokers, and the quit success rate and use of licensed treatments among those who made a quit attempt.

**Results:**

The success rate of quit attempts increased by 0.040% (95% CI 0.019; 0.062) for every 1% increase in the prevalence of e-cigarette use during a quit attempt. No clear evidence was found for an association between current e-cigarette use and the quit attempt rate (*B*_adj_ = 0.008 [95% CI −0.045; 0.061]) or overall quit rate (*B*_adj_ = 0.063 [−0.031; 0.158]); or between use of e-cigarettes during a quit attempt and the overall quit rate (*B*_adj_ = 0.030 [−0.054; 0.114]), use of prescription medication (varenicline/bupropion/nicotine replacement therapy [NRT]: *B*_adj_ = −0.036 [−0.175; 0.102]), or use of over-the-counter NRT (*B*_adj_ = −0.052 [−0.120; 0.015]). There was no clear evidence this pattern of associations has changed substantially over time.

**Conclusions:**

Changes in the prevalence of e-cigarette use in England through 2022 have been positively associated with the success rate of quit attempts but not clearly associated with the quit attempt rate, overall quit rate, or use of licensed smoking cessation treatments.

**Implications:**

If the association between the increase in e-cigarette use and the quit success rate is causal, then the use of e-cigarettes in quit attempts has helped in the region of 30 000 to 50 000 additional smokers in England to successfully quit each year since they became popular in 2013, over and above the number who were quitting before the advent of e-cigarettes.

## Introduction

The e-cigarette market is rapidly evolving, and there have been notable shifts in the types of devices (generally referred to as e-cigarettes, vapes, or nicotine vaping products) being used over recent years.^1,2^ England has been unusual in attempts to balance the opportunities and risks from e-cigarettes,^3–6^ on the basis of observational and experimental evidence that they are both effective for helping people to stop smoking^7–11^ and substantially less harmful than smoking tobacco.^6^ It is therefore critical from a national and international perspective to continue to evaluate e-cigarettes’ impact on key quitting outcomes, and whether use is helping or harming public health (including indirect impacts such as using e-cigarettes instead of other medically licensed cessation aids) as the market develops.

E-cigarettes entered the market in England in 2005, but there was no significant uptake until 2011.^7,12^ Early e-cigarettes were disposable devices (sometimes referred to as “cigalikes”) designed to mimic the look and feel of cigarettes and to be discarded when they ran out of charge or e-liquid. Products have evolved substantially over the past decade, starting with rechargeable devices with refillable tanks and more modifiability (eg, adjustable power), then more recently pod-style devices (eg, JUUL) and new disposables (eg, Puff Bar, Elf Bar, Geek Bar) which use nicotine salts rather than free-base nicotine.^1,2,6^ These newer products deliver higher levels of nicotine^13–15^ and, as a result, may be more effective for helping people stop smoking.^16,17^ New disposable products have rapidly become popular in England over the last 2 years, particularly among young adults.^2^

A “living” Cochrane review regularly synthesizes evidence from randomized controlled trials (RCTs) to provide up-to-date estimates of the effectiveness of e-cigarettes for smoking cessation.^10^ The most recent iteration concluded there was high-certainty evidence that using an e-cigarette increases quit rates. However, RCT evidence is limited in terms of generalizability, as most e-cigarette use does not involve engagement with health professionals (the number of smokers who use e-cigarettes in a quit attempt is much higher than the number who seek support from health professionals working within stop smoking services^12^). Thus, it is important to also consider the latest observational evidence to provide triangulation on the true effect size and any changes over time.^18^

The Smoking Toolkit Study, a monthly household survey representative of adults in England, has been measuring e-cigarette use and smoking cessation behaviors since 2006—providing a unique opportunity to assess the impact of changes in the prevalence of e-cigarette use on key population-level measures of quitting among smokers. We previously published time-series analyses up to 2015 and subsequently 2017.^7,8^ Our results established that population-level changes in e-cigarette use in England were positively associated with overall quit rates but not clearly associated with quit attempts, mean cigarette consumption (ie, smoking reduction in people who continue to smoke), or use of other quitting aids (except nicotine replacement therapy [NRT] on prescription, where the association was negative). There were no notable differences in the pattern of results up to 2017 compared with 2015. The current paper extends these time-series analyses for a further 23 quarters (up to the end of 2022), offering three distinct benefits. First, the longer time series provides increased power to identify and estimate associations between e-cigarette use and key outcomes, should they exist. Secondly, it provides the opportunity to assess whether or not associations have remained stable over a period that has seen substantial changes in types of e-cigarette devices being used. Finally, the results provide up-to-date effect sizes for use by policy makers, to ensure decision making continues to be informed by current evidence.

We aimed to address the following research questions:

1. What is the association between the prevalence of e-cigarette use among current smokers and the prevalence of:
  a. quit attempts among past-year smokers;
  b. cessation among past-year smokers (ie, overall quit rate)?
2. What is the association between the prevalence of e-cigarette use in a quit attempt among past-year smokers and the prevalence of:
  a. quit success among past-year smokers who made a quit attempt (ie, the proportion of smokers who tried to quit who quit successfully);
  b. cessation among past-year smokers (ie, the proportion of all smokers who quit successfully—which we refer to as the “overall quit rate”);
  c. use of licensed smoking cessation treatments (any prescription treatment, NRT on prescription, and NRT bought over the counter) among past-year smokers who made a quit attempt?
3. Have these associations changed over time?

We included both quit success and the overall quit rate because they provide different insights into associations with quitting. E-cigarettes likely primarily affect quit success within a quit attempt,^8,10,19^ which is why it is a key outcome and likely most sensitive. The overall quit rate depends on quit attempts and quit success, and may also reflect people stopping smoking outside of quit attempts. If quit attempts decline, increased quit success may not lead to population-level reductions in quitting. Similarly, if there is a change in stopping outside of quit attempts, then it could either lead to a further reduction in population effects of e-cigarettes on cessation if it declines or a further increase if it increases. Insofar that there is a causal impact on quit success, everything else being equal, one would expect to see also an impact on overall quit rate. But if there is less or little impact on attempts or cessation outside of quit attempts, then noise from these sources may obscure the association.

## Methods

### Preregistration

The analysis plan was preregistered on Open Science Framework (https://osf.io/gdfvz/). We made two amendments. First, we had planned to analyze data from November 2006 (the first wave of the Smoking Toolkit Study) through December 2022, but no data on the covariate mass media expenditure were available before 2007, so we amended our study period to January 2007 through December 2022. Second, in a sensitivity analysis restricted to data collected since the second quarter of 2017 (ie, data collected since our previous analysis^8^), the adjusted models were overparameterized and would not run, so we used the incremental policy index to adjust for tobacco control policies, rather than modeling each policy as a separate variable.

### Design

Data on the explanatory and outcome variables were drawn from the Smoking Toolkit Study, a monthly cross-sectional survey representative of adults in England. The study’s sampling methods are described in full elsewhere.^20^ Briefly, England is split into output areas, which are stratified by region and demographic characteristics before being randomly selected for inclusion on the interview list. Interviews are conducted in these selected areas until quotas based on working status, age, and gender are met. Comparisons with other national surveys and sales data indicate key variables such as sociodemographic characteristics, smoking prevalence, and cigarette consumption are nationally representative.^20,21^

Data were collected monthly through face-to-face computer-assisted interviews up to February 2020. However, social distancing restrictions under the Covid-19 pandemic meant no data were collected in March 2020, data from April 2020 onwards were collected via telephone, and the lower age bound for participation was increased from 16 to 18 years due to changes in consenting procedures. The telephone-based data collection relied upon the same combination of random location and quota sampling, and weighting approach as the face-to-face interviews and the two data collection modalities show good comparability.^22–24^

For the present study, we used data from past-year smokers surveyed between January 2007 and December 2022. Because the sample was restricted to people aged ≥18 years when data collection switched from face-to-face to telephone interviews, we excluded any participants aged 16–17 recruited before April 2020 for consistency (note that our previous papers^7,8^ included all participants ≥16 years). Responses were aggregated quarterly, providing 64 data points from Q1-2007 to Q4-2022.

Quarterly data on the covariate national government expenditure on tobacco control mass media were obtained from the Office for Health Improvement and Disparities (part of the Department of Health and Social Care).

### Measures

#### Smoking Status

Participants were asked which of the following best applies to them:

a) I smoke cigarettes (including hand-rolled) every day
b) I smoke cigarettes (including hand-rolled), but not every day
c) I do not smoke cigarettes at all, but I do smoke tobacco of some kind (eg, pipe, cigar, or shisha)
d) I have stopped smoking completely in the last year
e) I stopped smoking completely more than a year ago
f) I have never been a smoker (ie, smoked for a year or more)

Those who reported currently smoking cigarettes (responses a–b) were considered *current smokers*. Those who reported currently smoking cigarettes or having stopped smoking within the last year (responses a, b, or d) were considered *past-year smokers*. Those who reported having stopped smoking more than a year ago (response e) were excluded as our outcomes of interest were not assessed in this group.

#### Explanatory Variables

Current smokers were asked the following questions:

1. Which, if any, of the following are you currently using to help you cut down the amount you smoke?
2. Do you regularly use any of the following in situations when you are not allowed to smoke?
3. Can I check, are you using any of the following either to help you stop smoking, to help you cut down, or for any other reason at all?

- Response options for each question were: nicotine gum, nicotine replacement lozenges/tablets, nicotine replacement inhaler, nicotine replacement nasal spray, nicotine patch, electronic cigarette, nicotine mouth spray, other.

*Prevalence of use of e-cigarettes among current smokers* in each quarter was calculated as the number who answered “electronic cigarette” in response to any of the three questions above, divided by the number of cigarette smokers.

Past-year smokers who reported having made a quit attempt during the previous 12 months were asked the following question:

1. Which, if any, of the following did you try to help you stop smoking during the most recent serious quit attempt?

- Response options included a list of cessation aids (including e-cigarettes).

*Prevalence of use of e-cigarettes in a quit attempt among past-year smokers* in each quarter was calculated as the number who reported having used e-cigarettes in their most recent quit attempt, divided by the number of past-year smokers who reported having made a quit attempt.

Because questions assessing e-cigarette use were only introduced to the Smoking Toolkit Study survey in July 2009, and there was not significant uptake of e-cigarettes before 2011, the prevalence of use of e-cigarettes was estimated to be approximately 0.1% before July 2009, as in previous analyses.^8^

#### Outcomes

Past-year smokers were asked:

1. How many serious attempts to stop smoking have you made in the last 12 months? By serious attempt I mean you decided that you would try to make sure you never smoked again. Please include any attempt that you are currently making and please include any successful attempt made within the last year.

The *prevalence of quit attempts* in each quarter was calculated as the number who reported having made one or more quit attempt in the past 12 months divided by the number of past-year smokers.

Past-year smokers who reported having made a quit attempt are asked:

1. How long did your most recent serious quit attempt last before you went back to smoking?
2. Which, if any, of the following did you try to help you stop smoking during the most recent serious quit attempt?

- Response options included a list of cessation aids (including over-the-counter NRT, prescription NRT, varenicline, and bupropion).

The *quit success rate* in each quarter was calculated as the number who reported that they were still not smoking (in response to the question about how long the quit attempt lasted) divided by the number who reported having made a quit attempt. The *overall quit rate* in each quarter was calculated as the number who reported they were still not smoking following a quit attempt (in response to the question about how long the quit attempt lasted) divided by the number of past-year smokers.

The *prevalence of use of licensed smoking cessation treatments* in each quarter was analyzed as two variables distinguishing between prescription medication and over-the-counter treatments. In England, over-the-counter treatments refer to all products that can be brought in pharmacies and other shops (including supermarkets) without a prescription. These variables were calculated as the number who reported using (a) any treatment on prescription (NRT, varenicline, or bupropion) and (b) over-the-counter NRT divided by the number of past-year smokers who reported having made a quit attempt.

#### Covariates

Our analyses adjusted for government spending on national tobacco control mass media campaigns, tobacco control policies, and onset of the Covid-19 pandemic.

*Government mass media expenditure* per quarter (in £) was included to account for associations between level of expenditure on national tobacco control mass media campaigns and quitting activity.^25,26^ Data were obtained from the Office for Health Improvement and Disparities. Monthly totals include expenditure on TV, radio, print, cinema, and online advertisements across all tobacco control campaigns. In the months in which there was no campaign running and thus no campaign expenditure reported, campaign expenditure was entered as zero. This variable was log-transformed for analysis.

The following national *tobacco control policies* were included:

- the introduction of a smoking ban in July 2007^27^;
- the change in the minimum age of sale of cigarettes October 2007^28^;
- licensing of NRT for harm reduction in December 2009^29^;
- the move in commissioning of stop smoking services from the National Health Service (NHS) to local authorities (326 organizations responsible for public services and facilities in a particular district of England) in April 2013^30^;
- the publication of National Institute for Health and Care Excellence (NICE) guidance on harm reduction in June 2013^31^;
- the tobacco products directive in May 2016^32^;
- the publication of updated NICE guidance on treating tobacco dependence, which recommended e-cigarettes as a cessation aid in November 2021.^33^

There were no regional tobacco control policies implemented over the study period, although there were local tobacco control campaigns in some regions that were not controlled for in our analyses. For our primary analysis, we assumed a simple 1-quarter temporary pulse effect for each policy, as in previous analyses.^8^ We also ran sensitivity analyses where we modeled 2- and 3-quarter effects and an incremental policy index (coded 0 through 7, reflecting the number of these policies that had been implemented by each survey quarter). In addition, we ran a sensitivity analysis varying the date of the tobacco products directive to the end date of the implementation period (May 2017).^34^

Two variables related to the *timing of the Covid-19 pandemic* were included to account for increases in quit attempts and quit success during the pandemic.^22,23^ The first variable was coded 0 before the pandemic (ie, up to Q1-2020), 1 during the acute phase of the pandemic when there were restrictions on social interaction (ie, between Q2-2020 and Q2-2021^35^), and 0 after (ie, Q3-2021 onwards). The second was coded 0 before the pandemic (ie, up to Q1-2020) and 1 since (ie, Q2-2020 onwards) to account for the change in survey modality and long-term impact from the pandemic (from face-to-face to telephone interviews, respectively).

### Statistical Analysis

Data were analyzed using R v4.2.3. Data were aggregated quarterly (to boost the sample size for each data point, reduce noise and volatility in the time series, and match the interval of the mass media expenditure data) and weighted to match the population profile in England on age, social grade, region, tenure, ethnicity, and working status within sex. The dimensions are derived from a combination of the census, the Office for National Statistics and an annual random probability survey conducted for the National Readership Survey.^20^

#### Missing Data

Data on the prevalence of use of e-cigarettes among smokers have only been collected in the Smoking Toolkit Study since April 2011, although use during a recent quit attempt is available from July 2009. Thus, the prevalence of e-cigarette use among smokers between July 2009 and April 2011 was estimated from data on use during a quit attempt. Use of e-cigarettes among smokers between January 2007 and June 2009 were estimated to be 0.1% of smokers based on other surveys, which found their use to be extremely rare before 2009.^36,37^

Two waves of data were collected in March 2007, so these were combined. No data were collected in December 2008, so data for this period was calculated as an average of the month before and the month after. For a few months (May 2012, July 2012, September 2012, November 2012, January 2013, March 2013), data on e-cigarette use among smokers were not recorded. For these months, the average of the previous and next month was imputed.

For a number of months, mass media spending was effectively zero and was imputed as 0.1 to allow the analysis to run, as data were log-transformed to stabilize the variance. The same assumption was made for e-cigarette use where prevalence in the sample was zero.

#### ARIMAX Modeling

We used Autoregressive Integrated Moving Average with Exogeneous Input (ARIMAX)^38,39^ to estimate unadjusted and adjusted associations of e-cigarette use with quitting activity. ARIMAX is an extension of autoregressive integrated moving average analysis (ARIMA), which produces forecasts based upon prior values in the time-series analysis (AR terms) and the errors made by previous predictions (MA terms). Standard recommended procedures were used to select the ARIMAX models,^38,40^ as described in our previous papers.^7,8^ The series were log-transformed to stabilize the variance. Coefficients can be interpreted as estimates of the percentage change in the outcome of interest for every percentage increase in use of e-cigarettes and mass media, and absolute change as a consequence of tobacco control policies and the Covid-19 pandemic. We identified one outlier for current e-cigarette use (Q3-2019 = 0.174) and one for e-cigarette use during a quit attempt (Q4-2017 = 0.246); we report results with these outliers imputed (Q3-2019 = 0.147; Q4-2017 = 0.361) in the Supplementary Material. For all models, there was no violation of the Granger causality assumption, the assumption of normality was met, and autocorrelation terms were statistically significant and within the bounds of invertibility and stationarity.

Our primary analysis estimated associations of e-cigarette use with quitting activity over the entire study period (Q1-2007 to Q4-2022), providing an update on our previous estimates over a longer period.^7,8^ We ran four sensitivity analyses. First, we reran the adjusted analyses modeling tobacco control policies as 2- and 3-quarter pulse effects and as an incremental policy index (as described above). The second reran the adjusted analyses varying the timing of the tobacco products directive to the end date of the implementation period (rather than the start date, as described above). The third reran the models using the new data not included in our previous studies (Q2-2017 to Q4-2022) to explore whether the associations we had previously reported have changed over time. The fourth restricted the sample to 18- to 24-year olds to explore whether observed associations between e-cigarette use and quitting activity hold among young adults.

## Results

**Figure 1.**
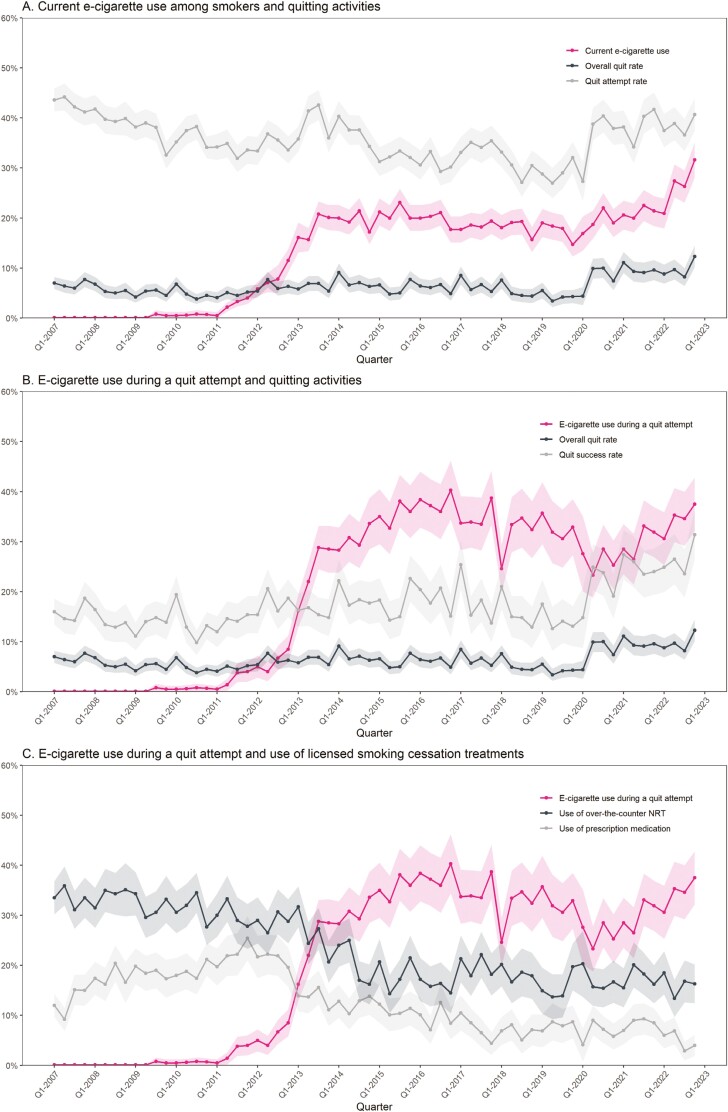
Quarterly prevalence of current e-cigarette use, use of e-cigarettes during a quit attempt, quitting activities, and use of licensed smoking cessation treatments. Lines represent weighted prevalence. Shaded bands represent 95% confidence intervals. *Prevalence of current e-cigarette use* among current smokers in each quarter is calculated as the number who report current use of e-cigarettes divided by the number of current smokers. *Prevalence of use of e-cigarettes during a quit attempt* among past-year smokers in each quarter is calculated as the number who report having used e-cigarettes in their most recent quit attempt, divided by the number of past-year smokers who report having made a quit attempt. The *quit attempt rate* in each quarter is calculated as the number who report having made one or more quit attempts in the past 12 months divided by the number of past-year smokers. The *overall quit rate* in each quarter is calculated as the number who report they are still not smoking following a quit attempt divided by the number of past-year smokers. The *quit success rate* in each quarter was calculated as the number who report that they are still not smoking divided by the number of past-year smokers who report having made a quit attempt. The prevalence of *use of licensed smoking cessation treatments* in each quarter is calculated as the number who report using (a) any prescription medication (NRT, varenicline, or bupropion) and (b) over-the-counter NRT divided by the number who report having made a quit attempt. NRT = nicotine replacement therapy.

Data were collected on 324 425 adults aged ≥18 taking part in the Smoking Toolkit Study who reported their smoking status. Of these, 67 548 were past-year smokers (weighted prevalence = 20.55%; 95% CI 20.41 to 20.69) and 62 439 were current smokers (weighted prevalence = 18.91%; 95% CI 18.77 to 19.04). Table 1 provides descriptive statistics for the prevalence of e-cigarette use, quitting activity, and use of licensed medication over the entire study period. Figure 1 shows the prevalence of these over time.

**Table 1.** Mean Quarterly Prevalence of E-cigarette Use, Quitting Activities, and Use of Licensed Smoking Cessation Treatments in England, Q1-2007 to Q4-2022

	Mean	SD	95% CI	
			Lower	Upper
Explanatory variables				
Current e-cigarette use^a^	13.19	9.30	10.55	15.84
E-cigarette use during a quit attempt^b^	20.45	15.30	13.28	27.62
Outcome variables				
Quit attempts^c^	35.71	4.31	35.14	36.28
Quit success^b^	17.54	4.60	16.89	18.19
Overall quit rate^c^	6.42	1.92	6.30	6.53
Use of licensed smoking cessation treatments^b^				
Prescription medications^d^	12.53	5.73	11.52	13.53
Over-the-counter NRT	23.43	7.23	21.83	25.03

^a^Among current smokers.

^b^Among past-year smokers who made a past-year quit attempt.

^c^Among past-year smokers.

^d^Varenicline, bupropion, or nicotine replacement therapy.

### Primary Analyses

Table 2 summarizes associations between the prevalence of e-cigarette use and quitting activity. In adjusted and unadjusted analyses, the data showed no clear association between the prevalence of e-cigarette use among current smokers and either attempts to quit smoking and overall quit rates. Similarly, the data showed no clear association between the prevalence of e-cigarette use during a quit attempt and overall quit rates. However, in adjusted analyses, the prevalence of e-cigarette use during a quit attempt was positively associated with the quit success rate, with every 1% rise in use associated with a 0.040% increase in the quit success rate.

**Table 2.** Estimated Percentage Point Changes in Quitting Activities as a Function of Current E-cigarette Use and E-cigarette Use During a Quit Attempt, Based on ARIMAX Models

	Quit attempt rate				Quit success rate				Overall quit rate			
	Percentage change per 1% change in the exposure	95% CI		*p*	Percentage change per 1% change in the exposure	95% CI		*p*	Percentage change per 1% change in the exposure	95% CI		*p*
		Lower	Upper			Lower	Upper			Lower	Upper	
Prevalence of current e-cigarette use												
Unadjusted^a^^,^^b^	0.003	−0.510	0.580	.907	—	—	—	—	0.080	−0.048	0.208	.219
Adjusted^a^^,^^b^	0.008	−0.045	0.061	.771	—	—	—	—	0.063	−0.031	0.158	.191
Prevalence of e-cigarette use during a quit attempt												
Unadjusted^a^^,^^b^	—	—	—	—	0.022	−0.074	0.118	.655	0.003	−0.125	0.131	.961
Adjusted^a^^,^^b^	—	—	—	—	0.040	0.019	0.062	<.001	0.030	−0.054	0.114	.480

Adjusted models control for government mass media expenditure, tobacco control policies, and the timing of the Covid-19 pandemic. The complete model output (including covariates) is provided in Supplementary Tables 1 (prevalence of current e-cigarette use) and 2 (prevalence of e-cigarette use during a quit attempt).

^a^Model = ARIMA(0,1,1)(0,0,0)_4_.

^b^No lag.

Table 3 summarizes associations between the prevalence of e-cigarette use during a quit attempt and use of licensed smoking cessation treatments. In adjusted and unadjusted analyses, the data showed no clear association between the prevalence of e-cigarette use during a quit attempt and either prescription medication or over-the-counter NRT.

**Table 3.** Estimated Percentage Point Changes in Use of Licensed Smoking Cessation Treatments as a Function of E-cigarette Use During a Quit Attempt, Based on ARIMAX Models

	Use of prescription medication				Use of over-the-counter NRT			
	Percentage change per 1% change in the exposure	95% CI		*p*	Percentage change per 1% change in the exposure	95% CI		*p*
		Lower	Upper			Lower	Upper	
Prevalence of e-cigarette use during a quit attempt								
Unadjusted^a,b^	−0.052	−0.192	0.912	.471	−0.051	−0.118	0.017	.140
Adjusted^a,b^	−0.036	−0.175	0.102	.607	−0.052	−0.120	0.015	.129

NRT = nicotine replacement therapy. Adjusted models control for government mass media expenditure, tobacco control policies, and the timing of the Covid-19 pandemic. The complete model output (including covariates) is provided in Supplementary Table 3.

^a^Model = ARIMA(0,1,1)(0,0,0)_4_.

^b^No lag.

### Sensitivity Analyses

The results of our primary analyses held when imputing outliers in the current e-cigarette series (Supplementary Tables 4–6); when modeling tobacco control policies as a 2-quarter pulse effect (Supplementary Tables 7–9), 3-quarter pulse effect (Supplementary Tables 10–12), or as an incremental policy index (Supplementary Tables 13–15); and when the timing of the tobacco products directive was modeled as the end date of the implementation period (Supplementary Tables 16–18).

When we restricted our study period to include new data not included in our previous studies (Q2-2017 to Q4-2022), estimates were less precise (indicated by wide 95% CIs; Supplementary Tables 19–21). Unadjusted and adjusted analyses showed a positive association between the prevalence of current e-cigarette use and the overall quit rate (Supplementary Table 19). The unadjusted analysis also showed a negative association between the prevalence of e-cigarette use during a quit attempt and the quit success rate, but this did not hold in the adjusted analysis, where the 95% CI (−0.959 to 0.450) overlapped the (positive) point estimate observed in our primary analysis (Supplementary Table 20). The analyses showed no clear association between the prevalence of e-cigarette use and rates of quit attempts (Supplementary Table 19) or use of licensed smoking cessation treatments (Supplementary Table 21).

When the sample was restricted to 18- to 24-year olds, unadjusted and adjusted analyses showed no clear association between the prevalence of e-cigarette use and any outcome of interest (Supplementary Tables 22–24). The point estimate for the adjusted association between the prevalence of e-cigarette use during a quit attempt and the quit success rate was smaller than was observed in our primary analysis of all adults, but in the same direction (*B*_adj_ = 0.028, 95% CI −0.010 to 0.067, *p* = .148; Supplementary Table 23).

## Discussion

Our results show a positive association between the prevalence of e-cigarette use in quit attempts and success rates of quit attempts among smokers in England after adjustment for a range of confounding variables. Each 1 percentage point increase in the use of e-cigarettes in a quit attempt was associated with a 0.040 percentage point increase in the quit success rate. This association was similar but smaller (0.028) and not statistically significant when the sample was restricted to young adults (18–24 years). No clear association was found between e-cigarette use and the prevalence of quit attempts, overall quit rates, or use of licensed smoking cessation treatments. There was no clear evidence this pattern of associations has changed substantially over time.

These results build on previous evidence, providing up-to-date estimates of associations between e-cigarette use and smoking cessation outcomes in England at the population level. Consistent with our previous analyses based on data up to 2015^7^ and 2017,^8^ we found that the success rate of quit attempts increased significantly as the proportion of smokers using e-cigarettes during a quit attempt increased. Methodological differences (specifically, the lower age limit for inclusion and covariates adjusted for) mean the results are not entirely comparable across the three studies. Nonetheless, the average effect for the association with quit success rates over the course of the study was relatively stable across analyses (2015: *B*_adj_ = 0.058 [95% CI 0.038 to 0.078]; 2017: *B*_adj_ = 0.060 [0.043 to 0.078]; 2022: *B*_adj_ = 0.040 [0.019 to 0.062]). The 2017 analysis also showed a significant association with overall quit rates. In our primary analysis, the point estimate for the adjusted association between current e-cigarette use and overall quit rate was similar to the 2017 estimate (0.063 vs. 0.054), but the 95% CI was wider, so it was not statistically significant. Our results for quit attempts and use of licensed smoking cessation treatments were in line with previous analyses, which also showed no clear evidence for an association with e-cigarette use.^7,8^

If the association identified in the current study between the increase in e-cigarette use and the quit success rate is causal, then every 1 percentage point increase in e-cigarette use in quit attempts would result in a 0.040 percentage point increase in quit success rate, other things being equal. Assuming that of the 5.82 million current smokers in England in 2022,^41,42^ 37% were attempting to quit^43^ and the prevalence of e-cigarette use in a quit attempt was 33% in that year,^12^ it is estimated that 710 622 (5 820 000 × 0.37 × 0.33) smokers used e-cigarettes during a quit attempt; this equates to approximately 28 400 (710 622 × 0.040) additional past-year smokers who report that they are no longer smoking as a consequence of e-cigarette use in a quit attempt in 2022. This is lower than the estimate we reported for 2017 (50 700),^8^ as a consequence of a reduction in the observed effect size (0.040 vs. 0.060) as well as in the population of smokers (5.82 million vs. 7 million). It is plausible that as smoking prevalence continues to decline, associations between e-cigarettes and quitting outcomes will weaken, as people who are helped to quit by e-cigarettes quit transition out of the remaining population of smokers, leaving those for whom e-cigarettes are less effective. However, it may also be the case that the development of more effective e-cigarettes may counter this effect. It also possible that increased uptake of current e-cigarettes and other harm reduction products (eg, nicotine pouches and heated tobacco products, which may be becoming more prevalent^44^) in the population could trigger quit attempts among smokers not using e-cigarettes, if it leads to increased media coverage about smoking (making smokers more concerned about their health) or further denormalization of smoking. Collectively, these analyses suggest that the use of e-cigarettes in quit attempts has helped in the region of 30 000 to 50 000 additional smokers in England to successfully quit each year since they have become popular. Between 2020 and 2021, smoking prevalence fell from 13.8% to 13.0% in England, which means there were approximately 360 000 fewer smokers, assuming a constant adult population of 44.77 million. The declines have been fairly similar annually since e-cigarettes became popular, which suggests e-cigarettes have contributed a sizeable proportion of the observed reduction in smoking prevalence each year since 2013. This calculation assumes that the primary mechanism by which e-cigarettes helps smokers transition to ex-smokers is to support a quit attempt. However, it may be that some smokers use e-cigarettes and end up cutting down and stopping without an intention to quit cigarettes and do not report a “quit attempt”.^45^ In our previous paper,^8^ we found a positive association between changes in current e-cigarette use and the overall quit rate, and used this to calculate an alternative estimate for the additional number who were helped by e-cigarettes directly or indirectly to quit smoking. This led to a larger figure of ~70 000 in 2017 due to the larger denominator (more smokers use e-cigarettes for any purpose than specifically in a quit attempt). Although the point estimate for the association between current e-cigarette use and the overall quit rate in our new analysis was similar to the 2017 figure (0.063 vs. 0.054, respectively), because it was not statistically significant, we have not updated this calculation.

Key strengths of this study include the nationally representative sample and long time series, offering insight into real-world impacts of e-cigarette use on smoking cessation behaviors. There were also several limitations. Some were common to our previous studies,^7,8^ including potential recall bias, the possibility of residual confounding, and limited generalizability to other countries with different tobacco control climates. A limitation specific to this analysis was the change in modality of data collection in April 2020 from face-to-face to telephone interviews. However, comparisons of data collected via the two methods indicate good comparability^22–24^ and our models accounted for this change through adjustment for the timing of the Covid-19 pandemic (which coincided with the switch to telephone interviews). In addition, we did not take into account the frequency (eg, daily vs. nondaily) or intensity (eg, cigarettes per day) of smoking; it is possible that there may be difference patterns of results across different types of smokers.

In conclusion, the rise in the prevalence of e-cigarette use among smokers in England has been associated with an increase in the success rate of quit attempts. There has been no clear association with changes in quit attempts, the overall quit rate, or use of licensed smoking cessation treatments. This pattern of associations has not changed substantially over time.

## Supplementary Material

Supplementary material is available at *Nicotine and Tobacco Research* online.

ntae007_suppl_Supplementary_Material

## Data Availability

All data used in these analyses are available on Open Science Framework (https://osf.io/gdfvz/).
