# Supplementary material for: Associations of Prevalence of E-cigarette Use With Quit Attempts, Quit Success, Use of Smoking Cessation Medication, and the Overall Quit Rate Among Smokers in England: A Time-Series Analysis of Population Trends 2007–2022
Source: Nicotine Tob Res. 2024 Jan 12;26(7):826–34. doi: 10.1093/ntr/ntae007 (PMC11190046; doi:10.1093/ntr/ntae007)
Supplement: ntae007_suppl_Supplementary_Material [file ntae007_suppl_supplementary_material.pdf]

Associations of prevalence of e-cigarette use with quit attempts, quit success, use of smoking cessation medication, and the overall quit rate in England: a time-series analysis of population trends 2006-2022

Supplementary material

Complete model output for primary analyses 2
Models with outliers imputed 5
Sensitivity analysis 1a: tobacco control policies modelled as 2 quarter pulse effects 8
Sensitivity analysis 1b: tobacco control policies modelled as 3 quarter pulse effects 11
Sensitivity analysis 1c: tobacco control policies modelled as an incremental policy index 14
Sensitivity analysis 2: timing of tobacco products directive changed to end of implementation period . . . 16
Sensitivity analysis 3: restricted to Q2 2017 onwards 19
Sensitivity analysis 4: restricted to 18-24 year-olds 22

1. Complete model output for primary analyses

Supplementary Table 1: Estimated percentage point changes in quitting activities as a function of current e-cigarette use, based on ARIMAX models

Unadjusted

	Quit attempt rate				Overall quit rate			
	Percentage change per 1% change in the exposure	95% CI Lower	95% CI Upper	*p*	Percentage change per 1% change in the exposure	95% CI Lower	95% CI Upper	*p*
Prevalence of current e-cigarette use	0.003	-0.510	0.580	0.907	0.080	-0.048	0.208	0.219
Model<br>Lag	ARIMA(0,1,1)(0,0,0)$_4$<br>No lag				ARIMA(0,1,1)(0,0,0)$_4$<br>No lag			

Adjusted

	Quit attempt rate				Overall quit rate			
	Percentage change per 1% change in the exposure	95% CI Lower	95% CI Upper	*p*	Percentage change per 1% change in the exposure	95% CI Lower	95% CI Upper	*p*
Prevalence of current e-cigarette use	0.008	-0.045	0.061	0.771	0.063	-0.031	0.158	0.191
Mass media	<0.001	-0.014	0.014	0.993	0.010	-0.032	0.053	0.639
	Total change due to the exposure	95% CI Lower	95% CI Upper	*p*	Total change due to the exposure	95% CI Lower	95% CI Upper	*p*
Smoking ban (temporary impact in Q3-2007)	-0.020	-0.150	0.111	0.768	0.005	-0.390	0.399	0.982
Increase in age of sale (temporary impact in Q4-2007)	-0.025	-0.154	0.103	0.698	0.271	-0.116	0.658	0.170
Licensing of NRT for harm reduction (temporary impact in Q4-2009)	-0.126	-0.246	-0.006	0.040	-0.171	-0.542	0.199	0.364
Move to local authority/ publication of NICE guidance on harm reduction (temporary impact in Q2-2013)	0.082	-0.045	0.208	0.205	0.092	-0.280	0.464	0.627
Tobacco products directive (temporary impact in the Q2-2016)	0.093	-0.029	0.216	0.135	-0.013	-0.386	0.360	0.946
Publication of updated NICE guidance on treating tobacco dependence, which recommended e-cigarettes as a cessation aid (temporary impact in Q4-2021)	0.068	-0.051	0.188	0.262	0.028	-0.362	0.417	0.890
Covid-19 pandemic – acute phase (step change between Q1-2020 and Q2-2021)	-0.128	-0.304	0.048	0.154	-0.032	-0.347	0.283	0.840
Covid-19 pandemic onset (step change between Q1-2020 and Q4-2022)	0.075	-0.178	0.328	0.561	0.549	0.135	0.962	0.009
Model<br>Lag for e-cigarettes<br>Lag for mass media	ARIMA(0,1,1)(0,0,0)$_4$<br>No lag<br>No lag				ARIMA(0,1,1)(0,0,0)$_4$<br>No lag<br>No lag			

Supplementary Table 2: Estimated percentage point changes in quitting activities as a function of e-cigarette use during a quit attempt, based on ARIMAX models

Unadjusted

	Quit success rate				Overall quit rate			
	Percentage change per 1% change in the exposure	95% CI Lower	95% CI Upper	*p*	Percentage change per 1% change in the exposure	95% CI Lower	95% CI Upper	*p*
Prevalence of e-cigarette use during a quit attempt	0.022	-0.074	0.118	0.655	0.003	-0.125	0.131	0.961
Model<br>Lag	$ARIMA(0,1,1)(0,0,0)_4$<br>No lag				$ARIMA(0,1,1)(0,0,0)_4$<br>No lag			

Adjusted

	Quit success rate				Overall quit rate			
	Percentage change per 1% change in the exposure	95% CI Lower	95% CI Upper	*p*	Percentage change per 1% change in the exposure	95% CI Lower	95% CI Upper	*p*
Prevalence of current e-cigarette use	0.040	0.019	0.062	**<0.001**	0.030	-0.054	0.114	0.480
Mass media	0.020	-0.010	0.051	0.194	0.012	-0.032	0.055	0.603
	Total change due to the exposure	95% CI Lower	95% CI Upper	*p*	Total change due to the exposure	95% CI Lower	95% CI Upper	*p*
Smoking ban (temporary impact in Q3-2007)	0.046	-0.291	0.383	0.789	0.008	-0.394	0.409	0.970
Increase in age of sale (temporary impact in Q4-2007)	0.319	-0.018	0.657	0.064	0.272	-0.122	0.666	0.176
Licensing of NRT for harm reduction (temporary impact in Q4-2009)	-0.041	-0.371	0.29	0.810	-0.164	-0.541	0.212	0.392
Move to local authority/ publication of NICE guidance on harm reduction (temporary impact in Q2-2013)	0.014	-0.315	0.342	0.936	0.097	-0.284	0.477	0.619
Tobacco products directive (temporary impact in the Q2-2016)	0.059	-0.271	0.388	0.727	-0.008	-0.388	0.371	0.966
Publication of updated NICE guidance on treating tobacco dependence, which recommended e-cigarettes as a cessation aid (temporary impact in Q4-2021)	-0.085	-0.44	0.27	0.639	0.018	-0.379	0.414	0.930
Covid-19 pandemic – acute phase (step change between Q1-2020 and Q2-2021)	-0.134	-0.331	0.062	0.181	-0.046	-0.359	0.266	0.772
Covid-19 pandemic onset (step change between Q1-2020 and Q4-2022)	0.442	0.284	0.599	**<0.001**	0.578	0.179	0.977	**0.005**
Model<br>Lag for e-cigarettes<br>Lag for mass media	$ARIMA(0,1,1)(0,0,0)_4$<br>No lag<br>No lag				$ARIMA(0,1,1)(0,0,0)_4$<br>No lag<br>No lag			

Supplementary Table 3: Estimated percentage point changes in use of licensed smoking cessation treatments as a function of e-cigarette use during a quit attempt, based on ARIMAX models

Unadjusted

	Use of prescription medication				Use of over-the-counter NRT			
	Percentage change per 1% change in the exposure	95% CI Lower	95% CI Upper	*p*	Percentage change per 1% change in the exposure	95% CI Lower	95% CI Upper	*p*
Prevalence of e-cigarette use during a quit attempt	-0.052	-0.192	0.912	0.471	-0.051	0.118	0.017	0.140
Model<br>Lag	$ARIMA(0,1,1)(0,0,0)_4$<br>No lag				$ARIMA(0,1,1)(0,0,0)_4$<br>No lag			

Adjusted

	Use of prescription medication				Use of over-the-counter NRT			
	Percentage change per 1% change in the exposure	95% CI Lower	95% CI Upper	*p*	Percentage change per 1% change in the exposure	95% CI Lower	95% CI Upper	*p*
Prevalence of current e-cigarette use	-0.036	-0.175	0.102	0.607	-0.052	-0.120	0.015	0.129
Mass media	-0.050	-0.088	-0.012	0.010	0.025	0.001	0.050	0.049
	Total change due to the exposure	95% CI Lower	95% CI Upper	*p*	Total change due to the exposure	95% CI Lower	95% CI Upper	*p*
Smoking ban (temporary impact in Q3-2007)	0.174	-0.201	0.549	0.362	-0.077	-0.320	0.166	0.535
Increase in age of sale (temporary impact in Q4-2007)	0.067	-0.303	0.438	0.721	-0.006	-0.246	0.235	0.964
Licensing of NRT for harm reduction (temporary impact in Q4-2009)	-0.062	-0.411	0.287	0.729	0.059	-0.176	0.293	0.623
Move to local authority/ publication of NICE guidance on harm reduction (temporary impact in Q2-2013)	-0.127	-0.478	0.224	0.479	-0.040	-0.284	0.205	0.751
Tobacco products directive (temporary impact in the Q2-2016)	-0.451	-0.802	-0.100	0.012	-0.092	-0.328	0.144	0.445
Publication of updated NICE guidance on treating tobacco dependence, which recommended e-cigarettes as a cessation aid (temporary impact in Q4-2021)	0.185	-0.179	0.549	0.320	-0.064	-0.309	0.181	0.610
Covid-19 pandemic – acute phase (step change between Q1-2020 and Q2-2021)	-0.135	-0.565	0.295	0.538	0.035	-0.178	0.247	0.750
Covid-19 pandemic onset (step change between Q1-2020 and Q4-2022)	-0.331	-0.914	0.253	0.267	0.001	-0.283	0.286	0.993
Model<br>Lag for e-cigarettes<br>Lag for mass media	$ARIMA(0,1,1)(0,0,0)_4$<br>No lag<br>No lag				$ARIMA(0,1,1)(0,0,0)_4$<br>No lag<br>No lag			

2. Models with outliers imputed

Supplementary Table 4: Estimated percentage point changes in quitting activities as a function of current e-cigarette use, based on ARIMAX models – outliers imputed

Adjusted	Quit attempt rate				Overall quit rate			
	Percentage change per 1% change in the exposure	95% CI Lower	95% CI Upper	*p*	Percentage change per 1% change in the exposure	95% CI Lower	95% CI Upper	*p*
Prevalence of current e-cigarette use	0.010	-0.043	0.064	0.701	0.063	-0.032	0.158	0.194
Mass media	<0.001	-0.014	0.014	0.985	0.010	-0.032	0.052	0.642
	Total change due to the exposure	95% CI Lower	95% CI Upper	*p*	Total change due to the exposure	95% CI Lower	95% CI Upper	*p*
Smoking ban (temporary impact in Q3-2007)	-0.020	-0.150	0.110	0.765	0.004	-0.390	0.398	0.984
Increase in age of sale (temporary impact in Q4-2007)	-0.026	-0.154	0.103	0.697	0.270	-0.117	0.658	0.171
Licensing of NRT for harm reduction (temporary impact in Q4-2009)	-0.126	-0.246	-0.006	0.040	-0.171	-0.542	0.199	0.364
Move to local authority/ publication of NICE guidance on harm reduction (temporary impact in Q2-2013)	0.082	-0.044	0.208	0.204	0.092	-0.280	0.464	0.627
Tobacco products directive (temporary impact in the Q2-2016)	0.094	-0.028	0.216	0.133	-0.013	-0.386	0.360	0.945
Publication of updated NICE guidance on treating tobacco dependence, which recommended e-cigarettes as a cessation aid (temporary impact in Q4-2021)	0.069	-0.051	0.188	0.261	0.028	-0.362	0.417	0.889
Covid-19 pandemic – acute phase (step change between Q1-2020 and Q2-2021)	-0.128	-0.304	0.047	0.152	-0.032	-0.347	0.284	0.843
Covid-19 pandemic onset (step change between Q1-2020 and Q4-2022)	0.075	-0.176	0.326	0.559	0.551	0.137	0.965	0.009
Model	$ARIMA(0,1,1)(0,0,0)_4$				$ARIMA(0,1,1)(0,0,0)_4$			
Lag for e-cigarettes	No lag				No lag			
Lag for mass media	No lag				No lag			

Supplementary Table 5: Estimated percentage point changes in quitting activities as a function of e-cigarette use during a quit attempt, based on ARIMAX models – outliers imputed

Adjusted

	Quit success rate				Overall quit rate			
	Percentage change per 1% change in the exposure	95% CI Lower	95% CI Upper	*p*	Percentage change per 1% change in the exposure	95% CI Lower	95% CI Upper	*p*
E-cigarette use during a quit attempt	0.041	0.019	0.062	**<0.001**	0.038	-0.046	0.121	0.377
Mass media	0.021	-0.010	0.052	0.182	0.012	-0.032	0.055	0.599
	Total change due to the exposure	95% CI Lower	95% CI Upper	*p*	Total change due to the exposure	95% CI Lower	95% CI Upper	*p*
Smoking ban (temporary impact in Q3-2007)	0.048	-0.289	0.384	0.781	0.009	-0.392	0.409	0.965
Increase in age of sale (temporary impact in Q4-2007)	0.321	-0.015	0.657	0.061	0.273	-0.120	0.667	0.173
Licensing of NRT for harm reduction (temporary impact in Q4-2009)	-0.040	-0.370	0.290	0.813	-0.166	-0.542	0.209	0.385
Move to local authority/ publication of NICE guidance on harm reduction (temporary impact in Q2-2013)	0.013	-0.314	0.341	0.937	0.093	-0.287	0.473	0.631
Tobacco products directive (temporary impact in the Q2-2016)	0.059	-0.270	0.387	0.727	-0.009	-0.387	0.370	0.965
Publication of updated NICE guidance on treating tobacco dependence, which recommended e-cigarettes as a cessation aid (temporary impact in Q4-2021)	-0.085	-0.440	0.269	0.636	0.018	-0.377	0.414	0.929
Covid-19 pandemic – acute phase (step change between Q1-2020 and Q2-2021)	-0.134	-0.330	0.062	0.181	-0.044	-0.356	0.268	0.780
Covid-19 pandemic onset (step change between Q1-2020 and Q4-2022)	0.442	0.284	0.599	**<0.001**	0.578	0.180	0.976	**0.004**
Model	ARIMA(0,1,1)(0,0,0)$_4$				ARIMA(0,1,1)(0,0,0)$_4$			
Lag for e-cigarettes	No lag				No lag			
Lag for mass media	No lag				No lag			

Supplementary Table 6: Estimated percentage point changes in use of licensed smoking cessation treatments as a function of e-cigarette use during a quit attempt, based on ARIMAX models – outliers imputed

Adjusted

	Use of prescription medication				Use of over-the-counter NRT			
	Percentage change per 1% change in the exposure	95% CI		*p*	Percentage change per 1% change in the exposure	95% CI		*p*
E-cigarette use during a quit attempt	-0.036	-0.176	0.104	0.614	-0.047	-0.117	0.023	0.185
Mass media	-0.050	-0.088	-0.012	**0.009**	0.024	-0.001	0.049	0.055
	Total change due to the exposure	95% CI Lower	Upper	*p*	Total change due to the exposure	95% CI Lower	Upper	*p*
Smoking ban (temporary impact in Q3-2007)	0.174	-0.201	0.549	0.364	-0.077	-0.321	0.167	0.535
Increase in age of sale (temporary impact in Q4-2007)	0.067	-0.303	0.438	0.722	-0.005	-0.246	0.236	0.966
Licensing of NRT for harm reduction (temporary impact in Q4-2009)	-0.062	-0.411	0.288	0.729	0.058	-0.177	0.293	0.629
Move to local authority/ publication of NICE guidance on harm reduction (temporary impact in Q2-2013)	-0.127	-0.479	0.224	0.478	-0.043	-0.288	0.202	0.730
Tobacco products directive (temporary impact in the Q2-2016)	-0.451	-0.802	-0.100	**0.012**	-0.092	-0.329	0.145	0.446
Publication of updated NICE guidance on treating tobacco dependence, which recommended e-cigarettes as a cessation aid (temporary impact in Q4-2021)	0.185	-0.179	0.550	0.319	-0.064	-0.309	0.182	0.612
Covid-19 pandemic – acute phase (step change between Q1-2020 and Q2-2021)	-0.135	-0.565	0.295	0.538	0.035	-0.179	0.250	0.746
Covid-19 pandemic onset (step change between Q1-2020 and Q4-2022)	-0.330	-0.912	0.253	0.268	0.002	-0.286	0.290	0.989
Model	$ARIMA(0,1,1)(0,0,0)_4$				$ARIMA(0,1,1)(0,0,0)_4$			
Lag for e-cigarettes	No lag				No lag			
Lag for mass media	No lag				No lag			

3. Sensitivity analysis 1a: tobacco control policies modelled as 2 quarter pulse effects

Supplementary Table 7: Estimated percentage point changes in quitting activities as a function of current e-cigarette use, based on ARIMAX models – 2 quarter pulse

Adjusted

	Quit attempt rate				Overall quit rate			
	Percentage change per 1% change in the exposure	95% CI Lower	95% CI Upper	*p*	Percentage change per 1% change in the exposure	95% CI Lower	95% CI Upper	*p*
Prevalence of current e-cigarette use	0.002	-0.050	0.054	0.948	0.057	-0.028	0.142	0.191
Mass media	0.001	-0.012	0.014	0.857	0.016	-0.029	0.062	0.486
	Total change due to the exposure	95% CI Lower	Upper	*p*	Total change due to the exposure	95% CI Lower	Upper	*p*
Smoking ban (temporary impact in Q3-2007)	-0.025	-0.129	0.079	0.636	0.070	-0.246	0.386	0.663
Increase in age of sale (temporary impact in Q4-2007)	0.014	-0.090	0.117	0.794	0.264	-0.048	0.575	0.097
Licensing of NRT for harm reduction (temporary impact in Q4-2009)	-0.113	-0.217	-0.008	**0.034**	0.087	-0.203	0.376	0.556
Move to local authority/ publication of NICE guidance on harm reduction (temporary impact in Q2-2013)	0.152	0.049	0.255	**0.004**	0.086	-0.197	0.369	0.550
Tobacco products directive (temporary impact in the Q2-2016)	0.097	-0.019	0.212	0.100	0.061	-0.225	0.348	0.676
Publication of updated NICE guidance on treating tobacco dependence, which recommended e-cigarettes as a cessation aid (temporary impact in Q4-2021)	0.002	-0.102	0.106	0.969	-0.039	-0.351	0.273	0.806
Covid-19 pandemic – acute phase (step change between Q1-2020 and Q2-2021)	-0.141	-0.301	0.019	0.084	-0.064	-0.377	0.249	0.690
Covid-19 pandemic onset (step change between Q1-2020 and Q4-2022)	0.070	-0.186	0.327	0.592	0.583	0.197	0.970	**0.003**
Model	$ARIMA(0,1,1)(0,0,0)_4$				$ARIMA(0,1,1)(0,0,0)_4$			
Lag for e-cigarettes	No lag				No lag			
Lag for mass media	No lag				No lag			

Supplementary Table 8: Estimated percentage point changes in quitting activities as a function of e-cigarette use during a quit attempt, based on ARIMAX models – 2 quarter pulse

Adjusted

	Quit success rate				Overall quit rate			
	Percentage change per 1% change in the exposure	95% CI		*p*	Percentage change per 1% change in the exposure	95% CI		*p*
E-cigarette use during a quit attempt	0.049	0.027	0.071	<0.001	0.029	-0.051	0.108	0.477
Mass media	0.023	-0.007	0.053	0.139	0.017	-0.03	0.064	0.479
	Total change due to the exposure	95% CI Lower	Upper	*p*	Total change due to the exposure	95% CI Lower	Upper	*p*
Smoking ban (temporary impact in Q3-2007)	0.090	-0.175	0.355	0.505	0.069	-0.251	0.389	0.673
Increase in age of sale (temporary impact in Q4-2007)	0.269	0.003	0.535	0.048	0.260	-0.056	0.575	0.107
Licensing of NRT for harm reduction (temporary impact in Q4-2009)	0.144	-0.090	0.378	0.228	0.086	-0.212	0.383	0.572
Move to local authority/ publication of NICE guidance on harm reduction (temporary impact in Q2-2013)	-0.058	-0.289	0.174	0.626	0.095	-0.194	0.383	0.520
Tobacco products directive (temporary impact in the Q2-2016)	0.053	-0.267	0.374	0.744	0.067	-0.223	0.357	0.652
Publication of updated NICE guidance on treating tobacco dependence, which recommended e-cigarettes as a cessation aid (temporary impact in Q4-2021)	-0.067	-0.339	0.205	0.629	-0.049	-0.365	0.266	0.759
Covid-19 pandemic – acute phase (step change between Q1-2020 and Q2-2021)	-0.139	-0.342	0.064	0.180	-0.076	-0.388	0.237	0.635
Covid-19 pandemic onset (step change between Q1-2020 and Q4-2022)	0.445	0.276	0.614	<0.001	0.607	0.228	0.987	0.002
Model	$ARIMA(0,1,1)(0,0,0)_4$				$ARIMA(0,1,1)(0,0,0)_4$			
Lag for e-cigarettes	No lag				No lag			
Lag for mass media	No lag				No lag			

Supplementary Table 9: Estimated percentage point changes in use of licensed smoking cessation treatments as a function of e-cigarette use during a quit attempt, based on ARIMAX models – 2 quarter pulse

Adjusted

	Use of prescription medication				Use of over-the-counter NRT			
	Percentage change per 1% change in the exposure	95% CI		*p*	Percentage change per 1% change in the exposure	95% CI		*p*
E-cigarette use during a quit attempt	-0.046	-0.188	0.096	0.525	-0.057	-0.123	0.008	0.086
Mass media	-0.049	-0.088	-0.010	0.014	0.025	<0.001	0.050	0.054
	Total change due to the exposure	95% CI		*p*	Total change due to the exposure	95% CI		*p*
		Lower Upper				Lower	Upper	
Smoking ban (temporary impact in Q3-2007)	0.122	-0.19	0.434	0.442	-0.043	-0.241	0.155	0.671
Increase in age of sale (temporary impact in Q4-2007)	0.001	-0.310	0.313	0.994	0.002	-0.196	0.199	0.985
Licensing of NRT for harm reduction (temporary impact in Q4-2009)	0.002	-0.307	0.311	0.990	-0.012	-0.198	0.174	0.901
Move to local authority/ publication of NICE guidance on harm reduction (temporary impact in Q2-2013)	0.100	-0.210	0.410	0.528	0.057	-0.137	0.251	0.564
Tobacco products directive (temporary impact in the Q2-2016)	-0.451	-0.805	-0.097	0.012	-0.096	-0.335	0.143	0.431
Publication of updated NICE guidance on treating tobacco dependence, which recommended e-cigarettes as a cessation aid (temporary impact in Q4-2021)	0.042	-0.285	0.369	0.801	0.054	-0.146	0.254	0.597
Covid-19 pandemic – acute phase (step change between Q1-2020 and Q2-2021)	-0.166	-0.604	0.272	0.458	0.067	-0.146	0.281	0.537
Covid-19 pandemic onset (step change between Q1-2020 and Q4-2022)	-0.309	-0.902	0.285	0.308	-0.036	-0.314	0.242	0.802
Model	$ARIMA(0,1,1)(0,0,0)_4$				$ARIMA(0,1,1)(0,0,0)_4$			
Lag for e-cigarettes	No lag				No lag			
Lag for mass media	No lag				No lag			

4. Sensitivity analysis 1b: tobacco control policies modelled as 3 quarter pulse effects

Supplementary Table 10: Estimated percentage point changes in quitting activities as a function of current e-cigarette use, based on ARIMAX models – 3 quarter pulse

Adjusted

	Quit attempt rate				Overall quit rate			
	Percentage change per 1% change in the exposure	95% CI Lower	Upper	*p*	Percentage change per 1% change in the exposure	95% CI Lower	Upper	*p*
Prevalence of current e-cigarette use	-0.001	-0.056	0.055	0.985	0.069	-0.024	0.161	0.144
Mass media	0.002	-0.015	0.018	0.822	0.013	-0.033	0.060	0.567
	Total change due to the exposure	95% CI Lower	Upper	*p*	Total change due to the exposure	95% CI Lower	Upper	*p*
Smoking ban (temporary impact in Q3-2007)	0.007	-0.107	0.121	0.904	0.154	-0.156	0.464	0.330
Increase in age of sale (temporary impact in Q4-2007)	-0.007	-0.117	0.104	0.906	0.110	-0.182	0.402	0.459
Licensing of NRT for harm reduction (temporary impact in Q4-2009)	-0.071	-0.180	0.038	0.201	0.066	-0.187	0.32	0.608
Move to local authority/ publication of NICE guidance on harm reduction (temporary impact in Q2-2013)	0.063	-0.067	0.192	0.343	-0.048	-0.306	0.21	0.716
Tobacco products directive (temporary impact in the Q2-2016)	0.085	-0.054	0.224	0.231	-0.072	-0.343	0.198	0.601
Publication of updated NICE guidance on treating tobacco dependence, which recommended e-cigarettes as a cessation aid (temporary impact in Q4-2021)	0.017	-0.098	0.132	0.769	0.001	-0.295	0.297	0.993
Covid-19 pandemic – acute phase (step change between Q1-2020 and Q2-2021)	-0.115	-0.298	0.068	0.217	-0.035	-0.373	0.303	0.840
Covid-19 pandemic onset (step change between Q1-2020 and Q4-2022)	0.121	-0.183	0.425	0.435	0.549	0.119	0.978	**0.012**
Model	ARIMA(0,1,1)(0,0,0)$_4$				ARIMA(0,1,1)(0,0,0)$_4$			
Lag for e-cigarettes	No lag				No lag			
Lag for mass media	No lag				No lag			

Supplementary Table 11: Estimated percentage point changes in quitting activities as a function of e-cigarette use during a quit attempt, based on ARIMAX models – 3 quarter pulse

Adjusted

	Quit success rate				Overall quit rate			
	Percentage change per 1% change in the exposure	95% CI		*p*	Percentage change per 1% change in the exposure	95% CI		*p*
E-cigarette use during a quit attempt	0.053	0.029	0.076	<0.001	0.038	-0.050	0.125	0.399
Mass media	0.027	-0.004	0.058	0.091	0.014	-0.034	0.061	0.572
	Total change due to the exposure	95% CI<br>Lower Upper		*p*	Total change due to the exposure	95% CI<br>Lower	Upper	*p*
Smoking ban (temporary impact in Q3-2007)	0.171	-0.082	0.425	0.186	0.153	-0.161	0.467	0.339
Increase in age of sale (temporary impact in Q4-2007)	0.118	-0.137	0.372	0.365	0.103	-0.193	0.398	0.495
Licensing of NRT for harm reduction (temporary impact in Q4-2009)	0.080	-0.118	0.277	0.430	0.065	-0.195	0.325	0.622
Move to local authority/ publication of NICE guidance on harm reduction (temporary impact in Q2-2013)	-0.099	-0.295	0.096	0.320	-0.040	-0.304	0.225	0.769
Tobacco products directive (temporary impact in the Q2-2016)	0.047	-0.276	0.371	0.774	-0.071	-0.346	0.203	0.610
Publication of updated NICE guidance on treating tobacco dependence, which recommended e-cigarettes as a cessation aid (temporary impact in Q4-2021)	0.005	-0.257	0.266	0.971	-0.005	-0.305	0.295	0.973
Covid-19 pandemic – acute phase (step change between Q1-2020 and Q2-2021)	-0.111	-0.339	0.116	0.336	-0.046	-0.386	0.294	0.791
Covid-19 pandemic onset (step change between Q1-2020 and Q4-2022)	0.415	0.221	0.608	<0.001	0.576	0.150	1.003	0.008
Model	$ARIMA(0,1,1)(0,0,0)_4$				$ARIMA(0,1,1)(0,0,0)_4$			
Lag for e-cigarettes	No lag				No lag			
Lag for mass media	No lag				No lag			

Supplementary Table 12: Estimated percentage point changes in use of licensed smoking cessation treatments as a function of e-cigarette use during a quit attempt, based on ARIMAX models – 3 quarter pulse

Adjusted

	Use of prescription medication				Use of over-the-counter NRT			
	Percentage change per 1% change in the exposure	95% CI		*p*	Percentage change per 1% change in the exposure	95% CI		*p*
E-cigarette use during a quit attempt	-0.038	-0.178	0.101	0.592	-0.048	-0.120	0.023	0.184
Mass media	-0.041	-0.080	-0.002	**0.037**	0.023	-0.002	0.049	0.074
	Total change due to the exposure	95% CI Lower	Upper	*p*	Total change due to the exposure	95% CI Lower	Upper	*p*
Smoking ban (temporary impact in Q3-2007)	0.163	-0.143	0.469	0.297	-0.072	-0.263	0.119	0.462
Increase in age of sale (temporary impact in Q4-2007)	-0.085	-0.387	0.218	0.584	0.051	-0.136	0.238	0.595
Licensing of NRT for harm reduction (temporary impact in Q4-2009)	-0.016	-0.305	0.272	0.912	0.012	-0.153	0.177	0.886
Move to local authority/ publication of NICE guidance on harm reduction (temporary impact in Q2-2013)	-0.055	-0.345	0.236	0.712	-0.039	-0.225	0.147	0.683
Tobacco products directive (temporary impact in the Q2-2016)	-0.447	-0.793	-0.101	**0.011**	-0.092	-0.328	0.143	0.442
Publication of updated NICE guidance on treating tobacco dependence, which recommended e-cigarettes as a cessation aid (temporary impact in Q4-2021)	0.245	-0.057	0.547	0.112	-0.062	-0.251	0.127	0.522
Covid-19 pandemic – acute phase (step change between Q1-2020 and Q2-2021)	-0.085	-0.524	0.354	0.705	0.019	-0.210	0.249	0.870
Covid-19 pandemic onset (step change between Q1-2020 and Q4-2022)	-0.398	-0.987	0.190	0.185	0.019	-0.280	0.318	0.901
Model	ARIMA(0,1,1)(0,0,0)$_4$				ARIMA(0,1,1)(0,0,0)$_4$			
Lag for e-cigarettes	No lag				No lag			
Lag for mass media	No lag				No lag			

5. Sensitivity analysis 1c: tobacco control policies modelled as an incremental policy index

Supplementary Table 13: Estimated percentage point changes in quitting activities as a function of current e-cigarette use, based on ARIMAX models – policy index

Adjusted

	Quit attempt rate				Overall quit rate			
	Percentage change per 1% change in the exposure	95% CI Lower	95% CI Upper	*p*	Percentage change per 1% change in the exposure	95% CI Lower	95% CI Upper	*p*
Prevalence of current e-cigarette use	0.002	-0.055	0.059	0.935	0.060	-0.036	0.156	0.219
Mass media	<0.001	-0.015	0.016	0.954	0.008	-0.033	0.050	0.690
Policy index	0.007	-0.008	0.022	0.380	0.007	-0.036	0.051	0.742
	Total change due to the exposure	95% CI Lower	Upper	*p*	Total change due to the exposure	95% CI Lower	Upper	*p*
Covid-19 pandemic – acute phase (step change between Q1-2020 and Q2-2021)	-0.118	-0.290	0.054	0.179	-0.031	-0.340	0.279	0.846
Covid-19 pandemic onset (step change between Q1-2020 and Q4-2022)	0.121	-0.140	0.383	0.362	0.546	0.132	0.961	**0.010**
Model	ARIMA(0,1,1)(0,0,0)$_4$				ARIMA(0,1,1)(0,0,0)$_4$			
Lag for e-cigarettes	No lag				No lag			
Lag for mass media	No lag				No lag			

Supplementary Table 14: Estimated percentage point changes in quitting activities as a function of e-cigarette use during a quit attempt, based on ARIMAX models – policy index

Adjusted

	Quit success rate				Overall quit rate			
	Percentage change per 1% change in the exposure	95% CI		*p*	Percentage change per 1% change in the exposure	95% CI		*p*
E-cigarette use during a quit attempt	0.036	0.016	0.057	**0.001**	0.027	-0.061	0.115	0.548
Mass media	0.019	-0.012	0.051	0.226	0.009	-0.033	0.052	0.666
Policy index	0.010	-0.029	0.049	0.602	0.008	-0.037	0.052	0.737
	Total change due to the exposure	95% CI Lower	Upper	*p*	Total change due to the exposure	95% CI Lower	Upper	*p*
Covid-19 pandemic – acute phase (step change between Q1-2020 and Q2-2021)	-0.114	-0.309	0.081	0.252	-0.039	-0.348	0.269	0.803
Covid-19 pandemic onset (step change between Q1-2020 and Q4-2022)	0.423	0.273	0.573	**<0.001**	0.569	0.161	0.977	**0.006**
Model	ARIMA(0,1,1)(0,0,0)$_4$				ARIMA(0,1,1)(0,0,0)$_4$			
Lag for e-cigarettes	No lag				No lag			
Lag for mass media	No lag				No lag			

Supplementary Table 15: Estimated percentage point changes in use of licensed smoking cessation treatments as a function of e-cigarette use during a quit attempt, based on ARIMAX models – policy index

Adjusted

	Use of prescription medication				Use of over-the-counter NRT			
	Percentage change per 1% change in the exposure	95% CI		*p*	Percentage change per 1% change in the exposure	95% CI		*p*
E-cigarette use during a quit attempt	-0.041	-0.185	0.103	0.577	-0.051	-0.118	0.017	0.141
Mass media	-0.046	-0.086	-0.005	**0.027**	0.026	0.001	0.050	**0.042**
Policy index	-0.018	-0.061	0.025	0.413	-0.010	-0.037	0.017	0.483
	Total change due to the exposure	95% CI Lower	Upper	*p*	Total change due to the exposure	95% CI Lower	Upper	*p*
Covid-19 pandemic – acute phase (step change between Q1-2020 and Q2-2021)	-0.175	-0.613	0.262	0.432	0.041	-0.167	0.250	0.698
Covid-19 pandemic onset (step change between Q1-2020 and Q4-2022)	-0.267	-0.874	0.339	0.388	-0.005	-0.288	0.277	0.970
Model	$ARIMA(0,1,1)(0,0,0)_4$							
Lag for e-cigarettes	No lag							
Lag for mass media	No lag							

6. Sensitivity analysis 2: timing of tobacco products directive changed to end of implementation period

Supplementary Table 16: Estimated percentage point changes in quitting activities as a function of current e-cigarette use, based on ARIMAX models – end of TPD implementation

Adjusted	Quit attempt rate				Overall quit rate			
	Percentage change per 1% change in the exposure	95% CI Lower	95% CI Upper	*p*	Percentage change per 1% change in the exposure	95% CI Lower	95% CI Upper	*p*
Prevalence of current e-cigarette use	0.004	-0.047	0.055	0.889	0.064	-0.032	0.160	0.190
Mass media	0.002	-0.013	0.017	0.795	0.009	-0.033	0.052	0.661
	Total change due to the exposure	95% CI Lower	95% CI Upper	*p*	Total change due to the exposure	95% CI Lower	95% CI Upper	*p*
Smoking ban (temporary impact in Q3-2007)	-0.012	-0.148	0.124	0.867	0.001	-0.392	0.394	0.997
Increase in age of sale (temporary impact in Q4-2007)	-0.020	-0.154	0.114	0.769	0.268	-0.118	0.654	0.174
Licensing of NRT for harm reduction (temporary impact in Q4-2009)	-0.132	-0.260	-0.004	**0.043**	-0.172	-0.541	0.197	0.361
Move to local authority/ publication of NICE guidance on harm reduction (temporary impact in Q2-2013)	0.096	-0.037	0.228	0.156	0.091	-0.279	0.462	0.630
Tobacco products directive (temporary impact in the Q2-2016)	0.064	-0.068	0.195	0.342	-0.069	-0.447	0.309	0.719
Publication of updated NICE guidance on treating tobacco dependence, which recommended e-cigarettes as a cessation aid (temporary impact in Q4-2021)	0.068	-0.062	0.198	0.307	0.029	-0.359	0.417	0.884
Covid-19 pandemic – acute phase (step change between Q1-2020 and Q2-2021)	-0.099	-0.259	0.061	0.224	-0.029	-0.347	0.289	0.859
Covid-19 pandemic onset (step change between Q1-2020 and Q4-2022)	0.124	-0.113	0.361	0.305	0.543	0.120	0.965	**0.012**
Model	ARIMA(0,1,1)(0,0,0)$_4$				ARIMA(0,1,1)(0,0,0)$_4$			
Lag for e-cigarettes	No lag				No lag			
Lag for mass media	No lag				No lag			

Supplementary Table 17: Estimated percentage point changes in quitting activities as a function of e-cigarette use during a quit attempt, based on ARIMAX models – end of TPD implementation

Adjusted

	Quit success rate				Overall quit rate			
	Percentage change per 1% change in the exposure	95% CI		*p*	Percentage change per 1% change in the exposure	95% CI		*p*
E-cigarette use during a quit attempt	0.041	0.020	0.063	**<0.001**	0.030	-0.056	0.115	0.494
Mass media	0.020	-0.011	0.050	0.206	0.011	-0.032	0.054	0.623
	Total change due to the exposure	95% CI Lower	Upper	*p*	Total change due to the exposure	95% CI Lower	Upper	*p*
Smoking ban (temporary impact in Q3-2007)	0.047	-0.289	0.384	0.783	0.004	-0.396	0.404	0.985
Increase in age of sale (temporary impact in Q4-2007)	0.321	-0.016	0.658	0.062	0.269	-0.124	0.662	0.179
Licensing of NRT for harm reduction (temporary impact in Q4-2009)	-0.041	-0.371	0.289	0.808	-0.164	-0.539	0.211	0.391
Move to local authority/ publication of NICE guidance on harm reduction (temporary impact in Q2-2013)	0.009	-0.319	0.337	0.957	0.095	-0.284	0.475	0.623
Tobacco products directive (temporary impact in the Q2-2016)	-0.089	-0.418	0.240	0.598	-0.062	-0.447	0.323	0.752
Publication of updated NICE guidance on treating tobacco dependence, which recommended e-cigarettes as a cessation aid (temporary impact in Q4-2021)	-0.085	-0.439	0.270	0.640	0.019	-0.376	0.414	0.925
Covid-19 pandemic – acute phase (step change between Q1-2020 and Q2-2021)	-0.134	-0.330	0.062	0.180	-0.043	-0.359	0.273	0.791
Covid-19 pandemic onset (step change between Q1-2020 and Q4-2022)	0.436	0.279	0.594	**<0.001**	0.573	0.165	**0.981**	**0.006**
Model	ARIMA(0,1,1)(0,0,0)$_4$				ARIMA(0,1,1)(0,0,0)$_4$			
Lag for e-cigarettes	No lag				No lag			
Lag for mass media	No lag				No lag			

Supplementary Table 18: Estimated percentage point changes in use of licensed smoking cessation treatments as a function of e-cigarette use during a quit attempt, based on ARIMAX models – end of TPD implementation

Adjusted

	Use of prescription medication				Use of over-the-counter NRT			
	Percentage change per 1% change in the exposure	95% CI		*p*	Percentage change per 1% change in the exposure	95% CI		*p*
E-cigarette use during a quit attempt	-0.040	-0.180	0.101	0.580	-0.052	-0.121	0.018	0.144
Mass media	-0.045	-0.085	-0.004	0.030	0.025	0.001	0.050	0.043
	Total change due to the exposure	95% CI Lower	Upper	*p*	Total change due to the exposure	95% CI Lower	Upper	*p*
Smoking ban (temporary impact in Q3-2007)	0.163	-0.235	0.560	0.422	-0.077	-0.320	0.167	0.537
Increase in age of sale (temporary impact in Q4-2007)	0.061	-0.332	0.453	0.761	-0.005	-0.246	0.235	0.966
Licensing of NRT for harm reduction (temporary impact in Q4-2009)	-0.058	-0.431	0.315	0.761	0.059	-0.176	0.294	0.622
Move to local authority/ publication of NICE guidance on harm reduction (temporary impact in Q2-2013)	-0.123	-0.498	0.253	0.522	-0.043	-0.288	0.202	0.732
Tobacco products directive (temporary impact in the Q2-2016)	0.066	-0.308	0.441	0.729	-0.035	-0.272	0.202	0.772
Publication of updated NICE guidance on treating tobacco dependence, which recommended e-cigarettes as a cessation aid (temporary impact in Q4-2021)	0.206	-0.188	0.599	0.306	-0.065	-0.310	0.180	0.602
Covid-19 pandemic – acute phase (step change between Q1-2020 and Q2-2021)	-0.099	-0.54	0.343	0.662	0.036	-0.180	0.252	0.743
Covid-19 pandemic onset (step change between Q1-2020 and Q4-2022)	-0.329	-0.922	0.265	0.277	<0.001	-0.290	0.291	0.998
Model	$ARIMA(0,1,1)(0,0,0)_4$				$ARIMA(0,1,1)(0,0,0)_4$			
Lag for e-cigarettes	No lag				No lag			
Lag for mass media	No lag				No lag			

7. Sensitivity analysis 3: restricted to Q2 2017 onwards

Note: Adjusted models for this sensitivity analysis were overparameterised and would not run, so we used the incremental policy index from sensitivity analysis 1c to adjust for tobacco control policies (rather than modelling each policy as a separate variable)

Supplementary Table 19: Estimated percentage point changes in quitting activities as a function of current e-cigarette use, based on ARIMAX models – restricted to > quarter 2 2017

Unadjusted

	Quit attempt rate				Overall quit rate			
	Percentage change per 1% change in the exposure	95% CI Lower	95% CI Upper	*p*	Percentage change per 1% change in the exposure	95% CI Lower	95% CI Upper	*p*
Prevalence of current e-cigarette use	0.187	0.197	0.570	0.340	0.918	0.020	1.816	**0.045**
Model	ARIMA(0,1,1)(0,0,0)$_4$				ARIMA(0,1,1)(0,0,0)$_4$			
Lag	No lag				No lag			

Adjusted

	Quit attempt rate				Overall quit rate			
	Percentage change per 1% change in the exposure	95% CI		*p*	Percentage change per 1% change in the exposure	95% CI		*p*
Prevalence of current e-cigarette use	0.080	-0.252	0.412	0.638	1.087	0.248	1.927	**0.011**
Mass media	-0.010	-0.029	0.010	0.328	-0.016	-0.073	0.042	0.598
Policy index	0.017	-0.012	0.046	0.253	0.049	-0.059	0.157	0.375
	Total change due to the exposure	95% CI Lower Upper		*p*	Total change due to the exposure	95% CI Lower	95% CI Upper	*p*
Covid-19 pandemic – acute phase (step change between Q1-2020 and Q2-2021)	-0.221	-0.492	0.050	0.110	0.152	-0.180	0.484	0.371
Covid-19 pandemic onset (step change between Q1-2020 and Q4-2022)	-0.012	-0.340	0.315	0.942	0.272	-0.095	0.640	0.146
Model	ARIMA(0,1,1)(0,0,0)$_4$				ARIMA(0,1,1)(0,0,0)$_4$			
Lag for e-cigarettes	No lag				No lag			
Lag for mass media	No lag				No lag			

Supplementary Table 20: Estimated percentage point changes in quitting activities as a function of e-cigarette use during a quit attempt, based on ARIMAX models – restricted to > quarter 2 2017

Unadjusted

	Quit success rate				Overall quit rate			
	Percentage change per 1% change in the exposure	95% CI Lower	95% CI Upper	*p*	Percentage change per 1% change in the exposure	95% CI Lower	95% CI Upper	*p*
E-cigarette use during a quit attempt	-0.592	-1.180	-0.004	0.049	-0.602	-1.400	0.196	0.139
Model	ARIMA(0,1,1)(0,0,0)$_4$				ARIMA(0,1,1)(0,0,0)$_4$			
Lag	No lag				No lag			

Adjusted

	Quit success rate				Overall quit rate			
	Percentage change per 1% change in the exposure	95% CI Lower	95% CI Upper	*p*	Percentage change per 1% change in the exposure	95% CI Lower	95% CI Upper	*p*
E-cigarette use during a quit attempt	-0.255	-0.959	0.450	0.479	-0.290	-1.232	0.652	0.546
Mass media	-0.037	-0.080	0.006	0.092	-0.032	-0.092	0.027	0.284
Policy index	-0.011	-0.086	0.064	0.768	0.010	-0.092	0.112	0.851
	Total change due to the exposure	95% CI Lower	Upper	*p*	Total change due to the exposure	95% CI Lower	Upper	*p*
Covid-19 pandemic – acute phase (step change between Q1-2020 and Q2-2021)	-0.167	-0.497	0.163	0.320	-0.107	-0.548	0.334	0.634
Covid-19 pandemic onset (step change between Q1-2020 and Q4-2022)	0.520	0.111	0.930	0.013	0.547	-0.086	1.181	0.090
Model	ARIMA(0,1,1)(0,0,0)$_4$				ARIMA(0,1,1)(0,0,0)$_4$			
Lag for e-cigarettes	No lag				No lag			
Lag for mass media	No lag				No lag			

Supplementary Table 21: Estimated percentage point changes in use of licensed smoking cessation treatments as a function of e-cigarette use during a quit attempt, based on ARIMAX models – restricted to > quarter 2 2017

Unadjusted

	Use of prescription medication				Use of over-the-counter NRT			
	Percentage change per 1% change in the exposure	95% CI Lower	95% CI Upper	*p*	Percentage change per 1% change in the exposure	95% CI Lower	95% CI Upper	*p*
E-cigarette use during a quit attempt	-0.686	-1.595	0.222	0.139	-0.175	-0.619	0.270	0.441
Model<br>Lag	ARIMA(0,1,1)(0,0,0)$_4$<br>No lag				ARIMA(0,1,1)(0,0,0)$_4$<br>No lag			

Adjusted

	Use of prescription medication				Use of over-the-counter NRT			
	Percentage change per 1% change in the exposure	95% CI Lower	95% CI Upper	*p*	Percentage change per 1% change in the exposure	95% CI Lower	95% CI Upper	*p*
E-cigarette use during a quit attempt	-0.505	-1.435	0.425	0.287	-0.333	-0.921	0.254	0.266
Mass media	-0.080	-0.142	-0.018	**0.011**	0.031	-0.005	0.068	0.088
Policy index	0.040	-0.064	0.143	0.451	-0.016	-0.079	0.048	0.63
	Total change due to the exposure	95% CI Lower Upper		*p*	Total change due to the exposure	95% CI Lower	95% CI Upper	*p*
Covid-19 pandemic – acute phase (step change between Q1-2020 and Q2-2021)	-0.323	-0.915	0.270	0.286	-0.036	-0.242	0.170	0.732
Covid-19 pandemic onset (step change between Q1-2020 and Q4-2022)	-0.254	-0.983	0.476	0.496	-0.058	-0.191	0.076	0.398
Model<br>Lag for e-cigarettes<br>Lag for mass media	ARIMA(0,1,1)(0,0,0)$_4$<br>No lag<br>No lag				ARIMA(0,1,1)(0,0,0)$_4$<br>No lag<br>No lag			

8. Sensitivity analysis 4: restricted to 18-24 year-olds

Supplementary Table 22: Estimated percentage point changes in quitting activities as a function of current e-cigarette use, based on ARIMAX models – restricted to 18 to 24 year olds

Unadjusted

	Quit attempt rate				Overall quit rate			
	Percentage change per 1% change in the exposure	95% CI Lower	95% CI Upper	*p*	Percentage change per 1% change in the exposure	95% CI Lower	95% CI Upper	*p*
Prevalence of current e-cigarette use	0.037	-0.027	0.101	0.257	-0.048	-0.192	0.096	0.516
Model<br>Lag	$ARIMA(0,1,1)(0,0,0)_4$<br>No lag				$ARIMA(0,1,1)(0,0,0)_4$<br>No lag			

Adjusted

	Quit attempt rate				Overall quit rate			
	Percentage change per 1% change in the exposure	95% CI Lower	95% CI Upper	*p*	Percentage change per 1% change in the exposure	95% CI Lower	95% CI Upper	*p*
Prevalence of current e-cigarette use	0.030	-0.027	0.087	0.305	-0.017	-0.062	0.028	0.456
Mass media	-0.015	-0.040	0.009	0.225	0.017	-0.040	0.073	0.561
	Total change due to the exposure	95% CI Lower	Upper	*p*	Total change due to the exposure	95% CI Lower	Upper	*p*
Smoking ban (temporary impact in third quarter of 2007)	-0.065	-0.295	0.165	0.582	0.331	-0.283	0.944	0.291
Increase in age of sale (temporary impact in fourth quarter of 2007)	0.031	-0.195	0.257	0.787	-0.389	-1.002	0.225	0.214
Licensing of nicotine replacement therapy (NRT) for harm reduction (temporary impact in fourth quarter of 2009)	-0.18	-0.404	0.044	0.115	-0.482	-1.078	0.115	0.113
Move to local authority/ publication of NICE guidance on harm reduction (temporary impact in second quarter of 2013)	0.084	-0.135	0.304	0.451	0.313	-0.283	0.909	0.303
Tobacco control directive (temporary impact in the second quarter of 2016)	0.107	-0.112	0.326	0.339	0.169	-0.429	0.766	0.580
Publication of updated NICE guidance on treating tobacco dependence, which recommended e-cigarettes as a cessation aid (temporary impact in fourth quarter of 2021)	0.321	0.092	0.549	**0.006**	-0.060	-0.704	0.584	0.855
COVID pandemic (step change between the 1st quarter of 2020 and 2nd quarter of 2021)	0.061	-0.153	0.276	0.575	-0.274	-0.631	0.084	0.133
Data collection change (step change between the 1st quarter of 2020 and 4th quarter of 2022)	0.363	0.041	0.684	**0.027**	0.935	0.642	1.228	**<0.001**
Model<br>Lag for e-cigarettes<br>Lag for mass media	$ARIMA(0,1,1)(0,0,0)_4$<br>No lag<br>No lag				$ARIMA(0,1,1)(0,0,0)_4$<br>No lag<br>No lag			

Supplementary Table 23: Estimated percentage point changes in quitting activities as a function of e-cigarette use during a quit attempt, based on ARIMAX models – restricted to 18 to 24 year olds

Unadjusted

	Quit success rate				Overall quit rate			
	Percentage change per 1% change in the exposure	95% CI Lower	95% CI Upper	*p*	Percentage change per 1% change in the exposure	95% CI Lower	95% CI Upper	*p*
E-cigarette use during a quit attempt	-0.052	-0.178	0.074	0.419	-0.097	-0.235	0.042	0.172
Model	ARIMA(0,1,1)(0,0,0)$_4$				ARIMA(0,1,1)(0,0,0)$_4$			
Lag	No lag				No lag			

Adjusted

	Quit success rate				Overall quit rate			
	Percentage change per 1% change in the exposure	95% CI Lower	95% CI Upper	*p*	Percentage change per 1% change in the exposure	95% CI Lower	95% CI Upper	*p*
E-cigarette use during a quit attempt	0.028	-0.010	0.067	0.148	-0.022	-0.061	0.018	0.285
Mass media	-0.003	-0.057	0.051	0.915	0.014	-0.041	0.070	0.615
	Total change due to the exposure	95% CI Lower	Upper	*p*	Total change due to the exposure	95% CI Lower	95% CI Upper	*p*
Smoking ban (temporary impact in third quarter of 2007)	0.318	-0.273	0.909	0.291	0.310	-0.299	0.919	0.318
Increase in age of sale (temporary impact in fourth quarter of 2007)	-0.470	-1.061	0.121	0.119	-0.409	-1.018	0.200	0.188
Licensing of nicotine replacement therapy (NRT) for harm reduction (temporary impact in fourth quarter of 2009)	-0.396	-0.972	0.180	0.178	-0.492	-1.086	0.102	0.105
Move to local authority/ publication of NICE guidance on harm reduction (temporary impact in second quarter of 2013)	0.128	-0.447	0.704	0.662	0.322	-0.271	0.915	0.288
Tobacco control directive (temporary impact in the second quarter of 2016)	-0.009	-0.586	0.568	0.976	0.176	-0.419	0.771	0.562
Publication of updated NICE guidance on treating tobacco dependence, which recommended e-cigarettes as a cessation aid (temporary impact in fourth quarter of 2021)	-0.315	-0.937	0.307	0.321	-0.065	-0.706	0.576	0.841
COVID pandemic (step change between the 1st quarter of 2020 and 2nd quarter of 2021)	-0.352	-0.696	-0.008	0.045	-0.273	-0.628	0.082	0.132
Data collection change (step change between the 1st quarter of 2020 and 4th quarter of 2022)	0.658	0.380	0.936	<0.001	0.939	0.652	1.225	<0.001
Model	ARIMA(0,1,1)(0,0,0)$_4$				ARIMA(0,1,1)(0,0,0)$_4$			
Lag for e-cigarettes	No lag				No lag			
Lag for mass media	No lag				No lag			

Supplementary Table 24: Estimated percentage point changes in use of licensed smoking cessation treatments as a function of e-cigarette use during a quit attempt, based on ARIMAX models – restricted to 18 to 24 year olds

Unadjusted

	Use of prescription medication				Use of over-the-counter NRT			
	Percentage change per 1% change in the exposure	95% CI		*p*	Percentage change per 1% change in the exposure	95% CI		*p*
		Lower	Upper			Lower	Upper	
E-cigarette use during a quit attempt	-0.076	-0.212	0.059	0.271	-0.100	-0.243	0.043	0.170
Model	ARIMA(0,1,1)(0,0,0)$_4$				ARIMA(0,1,1)(0,0,0)$_4$			
Lag	No lag				No lag			

Adjusted

	Use of prescription medication				Use of over-the-counter NRT			
	Percentage change per 1% change in the exposure	95% CI		*p*	Percentage change per 1% change in the exposure	95% CI		*p*
		Lower	Upper			Lower	Upper	
E-cigarette use during a quit attempt	-0.073	-0.209	0.062	0.290	-0.107	-0.244	0.030	0.126
Mass media	0.049	-0.025	0.123	0.192	0.065	-0.021	0.152	0.137
	Total change due to the exposure	95% CI		*p*	Total change due to the exposure	95% CI		*p*
		Lower Upper				Lower	Upper	
Smoking ban (temporary impact in third quarter of 2007)	-0.127	-0.867	0.613	0.737	-0.196	-1.084	0.692	0.666
Increase in age of sale (temporary impact in fourth quarter of 2007)	0.251	-0.476	0.979	0.499	0.226	-0.651	1.104	0.613
Licensing of nicotine replacement therapy (NRT) for harm reduction (temporary impact in fourth quarter of 2009)	0.039	-0.676	0.755	0.914	0.134	-0.724	0.992	0.760
Move to local authority/ publication of NICE guidance on harm reduction (temporary impact in second quarter of 2013)	-0.197	-0.918	0.523	0.592	-0.086	-0.954	0.782	0.845
Tobacco control directive (temporary impact in the second quarter of 2016)	-0.563	-1.27	0.144	0.119	-0.496	-1.340	0.349	0.250
Publication of updated NICE guidance on treating tobacco dependence, which recommended e-cigarettes as a cessation aid (temporary impact in fourth quarter of 2021)	0.118	-0.635	0.87	0.759	-0.030	-0.928	0.869	0.949
COVID pandemic (step change between the 1st quarter of 2020 and 2nd quarter of 2021)	-0.279	-0.841	0.282	0.330	-0.236	-0.812	0.339	0.421
Data collection change (step change between the 1st quarter of 2020 and 4th quarter of 2022)	0.170	-0.532	0.872	0.635	0.287	-0.360	0.935	0.385
Model	ARIMA(0,1,1)(0,0,0)$_4$				ARIMA(0,1,1)(0,0,0)$_4$			
Lag for e-cigarettes	No lag				No lag			
Lag for mass media	No lag				No lag			
